# Natural History of Swiss Infants with Non-SCID T-cell Lymphopenia Detected by Newborn Screening: A Cohort Study

**DOI:** 10.1007/s10875-025-01945-4

**Published:** 2025-11-25

**Authors:** Maarja Soomann, Seraina Prader, Philipp K. A. Agyeman, Geraldine Blanchard-Rohner, Michael Buettcher, Christian R. Kahlert, Nicole Ritz, Aikaterini Theodoropoulou, Jana Pachlopnik Schmid, Johannes Trück

**Affiliations:** 1 Division of Immunology and the Children’s Research Center, University Children’s Hospital Zurich, University of Zurich, Zurich, Switzerland; 2 Division of Pediatric Infectious Disease, Department of Pediatrics, lnselspital, Bern University Hospital, University of Bern, Bern, Switzerland; 3 Unit of Immunology, Vaccinology and Rheumatology, Division of General Pediatrics, Department of Pediatrics, Gynecology and Obstetrics, Geneva University Hospitals, Geneva, Switzerland; 4 Pediatric Infectious Diseases, Department of Pediatrics, Children’s Hospital of Central Switzerland (KidZ), Lucerne Cantonal Hospital, Lucerne, Switzerland; 5 Faculty of Health Sciences and Medicine, Lucerne University, Lucerne, Switzerland; 6 Pediatric Pharmacology and Pharmacometrics Research Center, University Children’s Hospital Basel (UKBB), Basel, Switzerland; 7 Division of Infectious Diseases, Infection Prevention, Children’s Hospital of Eastern Switzerland, St. Gallen, Switzerland; 8 Division of Infectious Diseases, Infection Prevention and Travel Medicine, Children’s Cantonal Hospital of St. Gallen, St. Gallen, Switzerland; 9 Mycobacterial and Migrant Health Research, Department for Clinical Research, University Children’s Hospital Basel, University of Basel, Basel, Switzerland; 10 Pediatric Immunology-Rheumatology-Allergology Unit, Woman-Mother- Child Department, University Hospital of Lausanne, University of Lausanne, Lausanne, Switzerland

**Keywords:** Newborn screening, TREC, T-cell deficiency, T-cell lymphopenia, TCL, Combined immunodeficiency, Idiopathic T-cell lymphopenia, Genetics, Inborn errors of immunity

## Abstract

**Background:**

Newborn screening (NBS) by quantification of T-cell receptor excision circles (TREC) identifies a considerable number of infants with T-cell lymphopenia (TCL) other than severe combined immunodeficiency (SCID). While some of these children have well-defined inborn errors of immunity (IEI), many lack a clear genetic diagnosis, complicating their management and causing prognostic uncertainty.

**Objective:**

To characterize the natural history of non-SCID TCL detected through NBS in Swiss infants between 2019 and 2023.

**Methods:**

Clinical, genetic and laboratory data from all non-SCID TCL cases were extracted from the national NBS registry and analyzed.

**Results:**

Out of 435 985 screened infants, 42 patients were identified with non-SCID, non-congenital athymia TCL, without an obvious secondary cause. A clear genetic diagnosis of IEI was established in 20 (48%) patients. Infants with confirmed IEI had significantly lower total T-cell, CD4 + T-cell and recent thymic emigrant (RTE) counts on initial lymphocyte phenotyping. In contrast, those with an unclear genetic diagnosis despite full investigations demonstrated faster normalization of total T-cell counts (hazard ratio 5.2, 95% CI 1.9 to 14.5, *p* = 0.001). All infants with initial CD4 + T-cell < 0.3 × 10^9^/L showed minimal recovery of T-cell counts and remained on long-term prophylactic measures. All infants with an unclear genetic diagnosis despite investigations were able to discontinue prophylaxis at median age 6 months without experiencing opportunistic or severe infections.

**Conclusion:**

Infants with non-SCID TCL identified by NBS represent a heterogenous group, ranging from severe, persistent TCL to mild, transient lymphopenia. Management should be tailored based on individual immunological and genetic profiles.

**Supplementary Information:**

The online version contains supplementary material available at 10.1007/s10875-025-01945-4.

## Highlights

Initial T-cell counts and genetic diagnosis are key prognostic factors in newborn screening-identified non-SCID T-cell lymphopenia. Patients with milder lymphopenia and no genetic diagnosis are more likely to experience T-cell recovery.

**Supplementary Information:**

The online version contains supplementary material available at 10.1007/s10875-025-01945-4.

## Introduction

The introduction of T-cell receptor excision circle (TREC)-based assays [1] into national newborn screening (NBS) programs has revolutionized the early detection of severe combined immunodeficiency (SCID) globally, enabling timely intervention and significantly improving patient outcomes [2–4]. However, while TREC assays are effective in identifying SCID, they are not specific to this condition [2, 5]. These assays detect infants with low numbers of naïve T-cells, irrespective of the underlying cause, and thus also identify newborns with T-cell lymphopenia (TCL) who do not have typicnaival or leaky SCID [2, 5, 6]. In addition, false-positive TREC results are not uncommon, with some newborns showing low TREC levels despite normal naïve T-cell counts [2, 7].

The non-SCID TCL group is highly heterogeneous and includes infants with syndromic and non-syndromic combined immunodeficiencies, such as 22q11.2 deletion syndrome (22q11.2DS), CHARGE syndrome, or other defined IEI, as well as idiopathic TCL and secondary causes, including prematurity. The severity within these conditions can vary significantly; for instance, 22q11.2DS typically results in mild immunodeficiency but can also present as congenital athymia, a profound T-cell defect requiring thymus transplant (TT), rather than hematopoietic stem cell transplantation (HSCT), the standard treatment for SCID. While the management pathways for SCID and congenital athymia, despite their urgency and complexity, are relatively well-defined [8–11], the management of infants with TCL who do not have a clear indication for HSCT or TT is far less straightforward [6, 9, 12].

Current recommendations for managing patients detected by NBS primarily focus on SCID [10, 12], leaving a significant gap in guidance for non-SCID TCL [13, 14]. There is limited published data on the natural history and optimal management of these children, highlighting the need for more comprehensive research.

In January 2019, Switzerland implemented national NBS for SCID and severe TCL using simultaneous quantification of TREC and kappa-deleting recombination excision circles (KREC) [10, 15]. The objective of this study is to provide a detailed description of the natural course of patients with non-SCID, non-congenital athymia TCL without an obvious secondary cause, identified by NBS in Switzerland, thereby addressing existing gaps in the literature and contributing to more informed clinical management of these cases.

## Methods

This is an observational registry-based cohort study. All newborns in Switzerland with abnormal TREC and/or KREC results from their first dried blood spots (DBS) are prospectively recorded in a central registry at the University Children’s Hospital Zurich. The ImmunoIVD SPOT-it TREC & KREC Screening Kit was used for NBS [16]. Between January 2019 and December 2020, TREC < 10 and KREC < 6 copies/punch were considered abnormal; from January 2021 onwards, the cut-offs were adjusted to TREC < 6 and KREC < 4 copies/punch.

Data on screening results, immunophenotyping, genetic diagnosis, prophylactic and/or therapeutic measures, and clinical course were documented until the child was discharged from or lost to follow-up or deceased. The detailed confirmatory testing protocol has been previously published [10]. In brief, it entails a full blood count, lymphocyte phenotyping including naïve T-cells and quantification of immunoglobulins IgG, IgA and IgM in all referred patients as well as additional tests in those with a confirmed TCL. Data were retrieved from the registry in August 2024. Inclusion and exclusion criteria are shown in Table 1.

**Table 1:** Inclusion and exclusion criteria

Type	Criteria
Inclusion	1) an abnormal TREC and/or KREC result between January 2019 and December 2023; AND
	2) a peripheral blood total CD3 + T-cell count of < 2.5 × 10^9^/L or naïve CD4 + CD45RA + T-cell count of < 1.2 × 10^9^/L (lower margins of the local reference ranges) on initial immunological evaluation
Exclusion	1) diagnosis of SCID or congenital athymia; OR
	2) the presence of a likely secondary cause of TCL and either subsequent normalization of TREC levels and/or T-cell counts, or death before further measurements could be taken; OR
	3) lack of informed consent to participate in the study

*CD*, cluster of differentiation; *KREC*, kappa-deleting recombination excision circles; *SCID*, severe combined immunodeficiency; *TCL*, T-cell lymphopenia; *TREC*, T-cell receptor excision circles

In the Swiss NBS program, genetic testing is offered to all families of newborns with confirmed TCL without an apparent secondary cause. The sequence of genetic tests is determined by the presence of other clinical findings, with all patients undergoing at least comparative genomic hybridization array or 22q11.2-specific multiplex ligation-dependent probe amplification and whole-exome sequencing (WES) or trio WES unless a clear genetic diagnosis was established through initial testing. Patients were classified as having a clear genetic diagnosis of an IEI (henceforth: “clear genetic diagnosis”) if large rearrangements or pathogenic/likely pathogenic variants in genes associated with T-cell deficiencies were identified. Patients who underwent genetic testing, but either showed no abnormalities, had variants of unknown significance (VUS) in known T-cell deficiency genes, or had variants in genes not previously associated with T-cell deficiencies were considered to have no clear genetic diagnosis of an IEI (henceforth: “unclear genetic diagnosis”). Patients whose parents declined genetic testing were classified separately (henceforth: “no genetic testing”) and were excluded from comparisons based on genetic diagnosis.

Decisions regarding prophylactic measures were taken by physicians based on the patient’s immunophenotype and clinical course. Generally, *Pneumocystis jiroveci* pneumonia (PJP) prophylaxis was recommended for outpatients with CD4 + counts < 1.0 × 10^9^/L during the first 6 months of life. Prophylaxis was discontinued once the patient’s CD4 + counts exceeded 0.5 × 10^9^/L and age-appropriate levels of specific antibodies against tetanus toxoid, pneumococcal and *Haemophilus influenzae* serotype b polysaccharides were achieved following at least two doses of the respective vaccines, per the national vaccination schedule. These criteria were used to assess the safety of administering live vaccines. Fungal prophylaxis and seasonal passive respiratory syncytial virus (RSV) vaccinations were recommended for patients with CD4 + counts < 0.3 × 10^9^/L, while immunoglobulin replacement therapy (IgRT) was advised for patients with CD4 + counts < 0.3 × 10^9^/L or hypogammaglobulinemia.

In this study, patients were classified based on their initial CD4 + counts as having severe (< 0.3 × 10^9^/L), moderate (0.3 to 1.0 × 10^9^/L), or mild TCL (> 1.0 × 10^9^/L).

Data analysis was conducted using R version 4.2.2 [17], with the relevant packages and statistical methods listed in Tables E1 and E2 of the Online Repository. A *p*-value < 0.05 was considered statistically significant.

The study was approved by the Cantonal Ethics Commission of Zurich (2022–01029) and conducted in accordance with the Declaration of Helsinki. Parents or legal guardians gave informed consent per agreed protocol. The results are reported following the STROBE guidelines for observational studies [18].

## Results

### Demographics

Between January 2019 and December 2023, a total of 435 985 infants were screened. After excluding 11 patients diagnosed with SCID, 2 with congenital athymia, 8 with secondary TCL and 3 without informed consent, 42 patients were included in this study. Causes of secondary TCL included severe prematurity, critical illness due to congenital heart disease, pancytopenia, maternal immunosuppression and Langerhans cell histiocytosis. Additional details on patients with secondary TCL are provided in Table E3 of the Online Repository.

Approximately half of the patients were female (*n* = 22, 52%). Most (*n* = 36, 86%) were born at full term, while 6 (14%) were moderate to late preterm (32 to 35 weeks). None of the mothers had taken immunosuppressive medication during pregnancy. Gestational diabetes was reported in the mothers of 3 newborns (7%), all of whom reported good control with dietary measures alone. There were no cases of other types of diabetes.

Two of the identified children were first cousins, although there was no history of IEI in their families or other relatives. Only one family was known to be consanguineous.

### NBS Results

All but one patient had abnormal TREC values in their initial DBS. The exception was a patient with ataxia-telangiectasia (AT), who had KREC levels below the cut-off but TREC levels slightly above it (KREC 2, cutoff < 4, TREC 8, cutoff < 6 copies/punch).

**Fig. 1:**
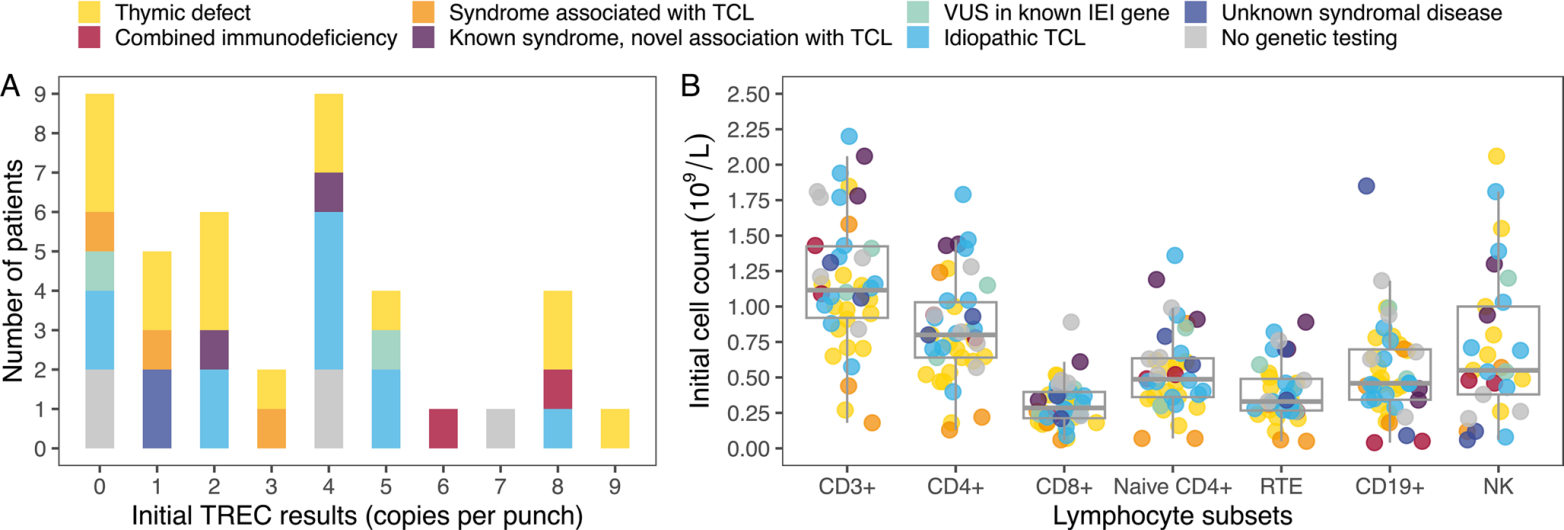
Distribution of initial TREC results (**A**), peripheral blood lymphocyte subset counts (**B**), and diagnostic subcategories. CD – cluster of differentiation; IEI – inborn errors of immunity; NK – natural killer; RTE – recent thymic emigrant; TCL – T-cell lymphopenia; TREC – T-cell receptor excision circle; VUS – variant of unclear significance

There was no consistent trend indicating that patients with clear genetic diagnoses, such as known thymic defects or combined immunodeficiencies, had lower initial TREC values. Unmeasurable TREC values were found both in patients with confirmed genetic diagnoses and in those with idiopathic TCL or only VUS in IEI genes. Exact TREC values by diagnostic group are shown in Fig. 1A.

The median age at the first immunological examination was 8 days. Details of these results are presented below.

### Genetic Evaluation

Genetic testing was declined by the families of five newborns (12%). Apart from higher CD8 + T-cell counts in those without genetic testing, there were no significant differences in the initial T-cell phenotyping results between those who underwent genetic testing and those who did not (Figure E1 in the Online Repository) indicating that the genetically tested patients were representative of the overall cohort.

A clear genetic diagnosis was established in 20 patients, with thymic defects being the most common finding. In 17 patients, the cause of their TCL remained unclear despite comprehensive genetic evaluation. The majority (*n* = 11, 65%) presented with isolated TCL and were classified as having idiopathic T-cell lymphopenia (iTCL). Details on the distribution of patients across diagnostic subcategories and final diagnoses are provided in Table 2.

**Table 2:** Genetic findings

Category	Subcategory	Final diagnosis	Count
*Clear genetic diagnosis*	Thymic defects	22q11.2 deletion syndrome	14
		FOXN1 haploinsufficiency	1
	Combined immunodeficiencies	Ataxia-teleangiectasia	2
	Syndromes associated with TCL	22q11.2 duplication syndrome	1
		SGPL1 deficiency	1
		Trisomy 21	1
*Unclear genetic diagnosis*	Known syndromes, novel association with TCL	KBG syndrome	1
		ZIMZ1 associated syndrome	1
	VUS in known IEI genes	VUS in LCK	1
		VUS in TBX1	1
	Idiopathic TCL		11
	Unknown syndromal disease with TCL		2
*No genetic testing*			5

*CID*, combined immunodeficiency; *IEI*, inborn errors of immunity; *TCL*, T-cell lymphopenia, *VUS*, variant of unknown significance

There was no significant association between initial TREC levels and the likelihood of establishing a clear genetic diagnosis (Figure E2 in the Online Repository). However, there was a trend indicating that patients with severe or moderate TCL were more likely to have a clear genetic diagnosis compared to those with mild TCL [χ^2^ (2, *N* = 37) = 6.16, *p* = 0.05].

### Initial Immunological Phenotype

The distribution of initial lymphocyte subset counts, along with the diagnostic subcategories of individual patients are shown in Fig. 1B. Total T-cell and T-cell subset counts varied widely among patients with thymic defects as well as those with idiopathic TCL. Both patients with ataxia-telangiectasia (AT) demonstrated very low CD19 + B-cell counts but only moderate T-cell lymphopenia in their initial investigations. T-cell proliferation testing results were available in three patients, with only one showing abnormal responses (a patient with iTCL). CD69 expression following mitogen stimulation was assessed in 23 patients, with abnormal results in two (one with FOXN1 haploinsufficiency and one with AT).

### Follow-up

At the time of the last follow-up, the majority of patients (*n* = 37, 88%) were alive, with a median age of 25 months (range: 2 to 59 months). Of the 5 deceased patients, four deaths were clearly attributable to causes unrelated to TCL (Table E4 in the Online Repository). Seven infants had been discharged from follow-up after normalization of their T-cell counts. Four families were lost to follow-up: two moved abroad, and two others declined further appointments despite persistently low T-cell counts. The remaining 26 patients continued to undergo immunological follow-up. The progression of CD4 + and recent thymic emigrant (RTE) counts in individual patients, categorized by diagnostic subcategory, is depicted in Figures E3 and E4 of the Online Repository.

**Fig. 2:**
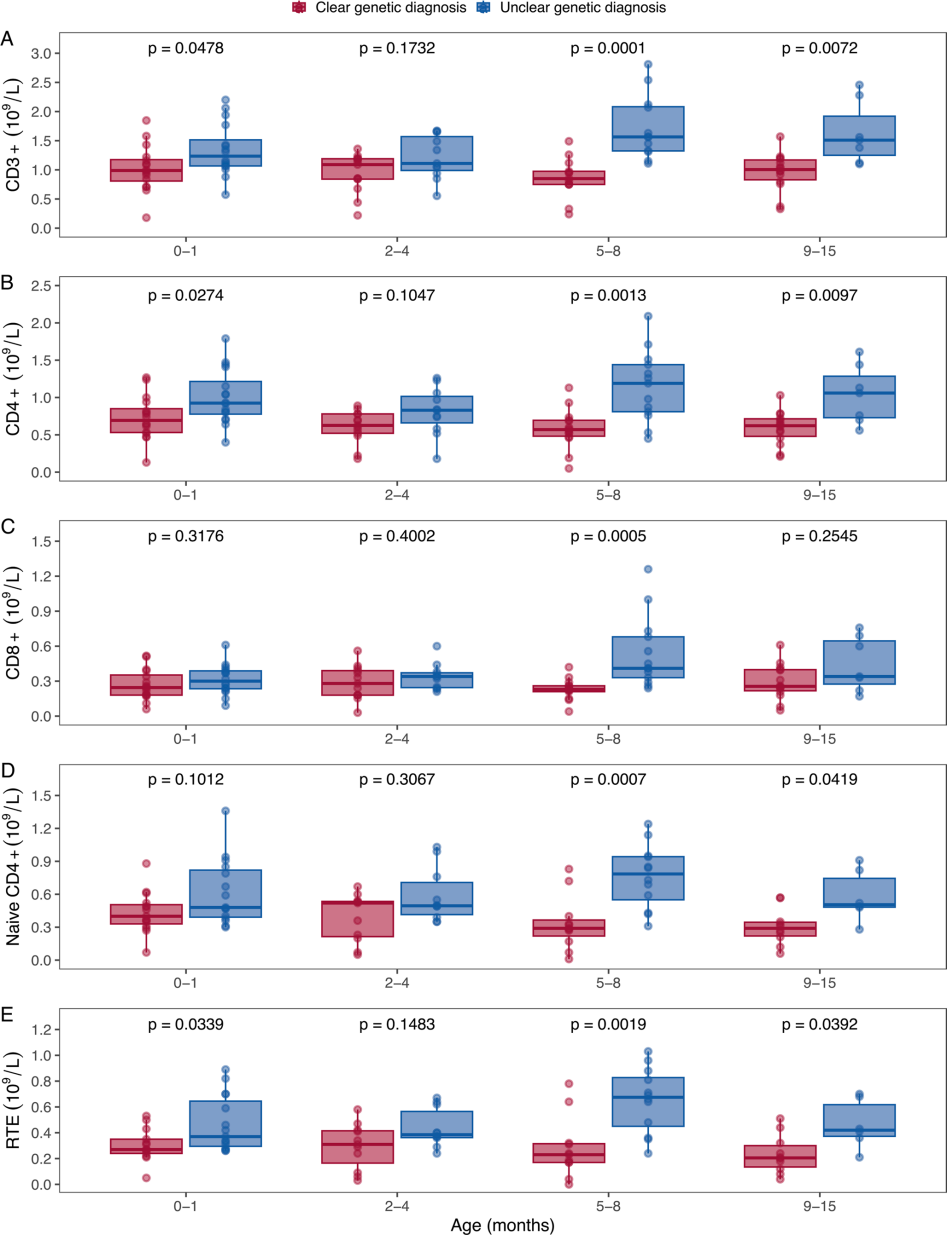
Comparison of total T-cell and T-cell subset counts in patients with a clear and unclear genetic diagnosis. Patients with a clear genetic diagnosis had lower CD3+, CD4 + and recent thymic emigrant (RTE) counts almost throughout the first year of life. Patients who underwent a thoracotomy during the first 15 months of life have been omitted

**Fig. 3:**
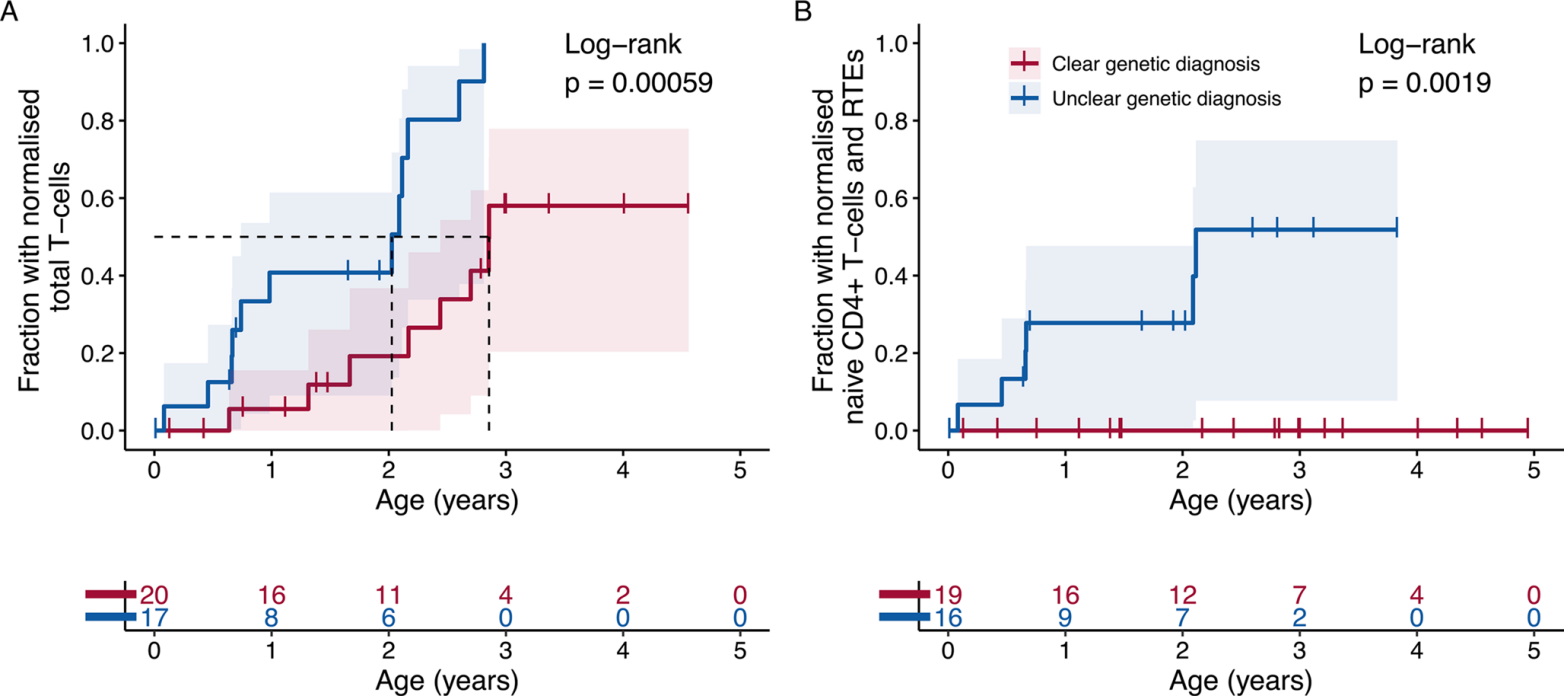
Time to normalization of total T-cells (**A**) and thymic output (**B**). Patients with an unclear genetic diagnosis exhibited a significantly faster normalization of their total T-cell counts (A) and thymic output (B), including naïve CD4 + T-cells and recent thymic emigrants (RTE), compared to those with a clear genetic diagnosis

Patients with a clear genetic diagnosis generally had lower total CD3 + and CD4 + T-cell, and RTE counts throughout the first year of life compared to those with an unclear genetic diagnosis (Fig. 2). By the last follow-up, more than half of the survivors (*n* = 22, 59%) had achieved normalization of their total CD3 + T-cell counts (median age 22 months; range: 1 to 34 months). In contrast, thymic output, had normalized in fewer than one-fifth of the survivors (*n* = 7, 19%) by a median age of 8 months (range: 1 to 25 months). Notably, patients with an unclear genetic diagnosis demonstrated a significantly faster normalization of their CD3 + T-cell counts (hazard ratio 5.2, 95% CI 1.9 to 14.5, *p* = 0.001) and thymic output, as shown in the time-to-event analysis (Fig. 3).

#### Patients with Severe TCL (CD4 + < 0.3 × 10^9^/L)

A clear genetic diagnosis was established in all three patients with severe TCL. The patient with 22q11.2 microduplication syndrome remained well under prophylactic measures, with a modest increase in CD4 + counts, although her RTE counts remained consistently low (previously published [19]). The patient with 22q11.2 deletion syndrome (22q11.2DS) suffered from serious congenital heart disease (CHD), requiring multiple thoracotomies. Although her CD4 + counts improved, stabilizing between 0.44 and 0.83 × 10^9^/L, she ultimately passed away at the age of 3 years due to chronic heart failure. The third patient, diagnosed with SGPL-1 deficiency, also suffered from chronic renal and adrenal insufficiency, which contributed to a complicated clinical course. Her CD4 + counts remained low, ranging between 0.05 and 0.28 × 10^9^/L, with an overall trend toward reduction (Figures E3C and E4C of the Online Repository). However, her TCL was not considered a major factor in her death at the age of 9 months.

#### Patients with Moderate TCL (CD4 + 0.3 to 1.0 × 10^9^/L)

**Fig. 4:**
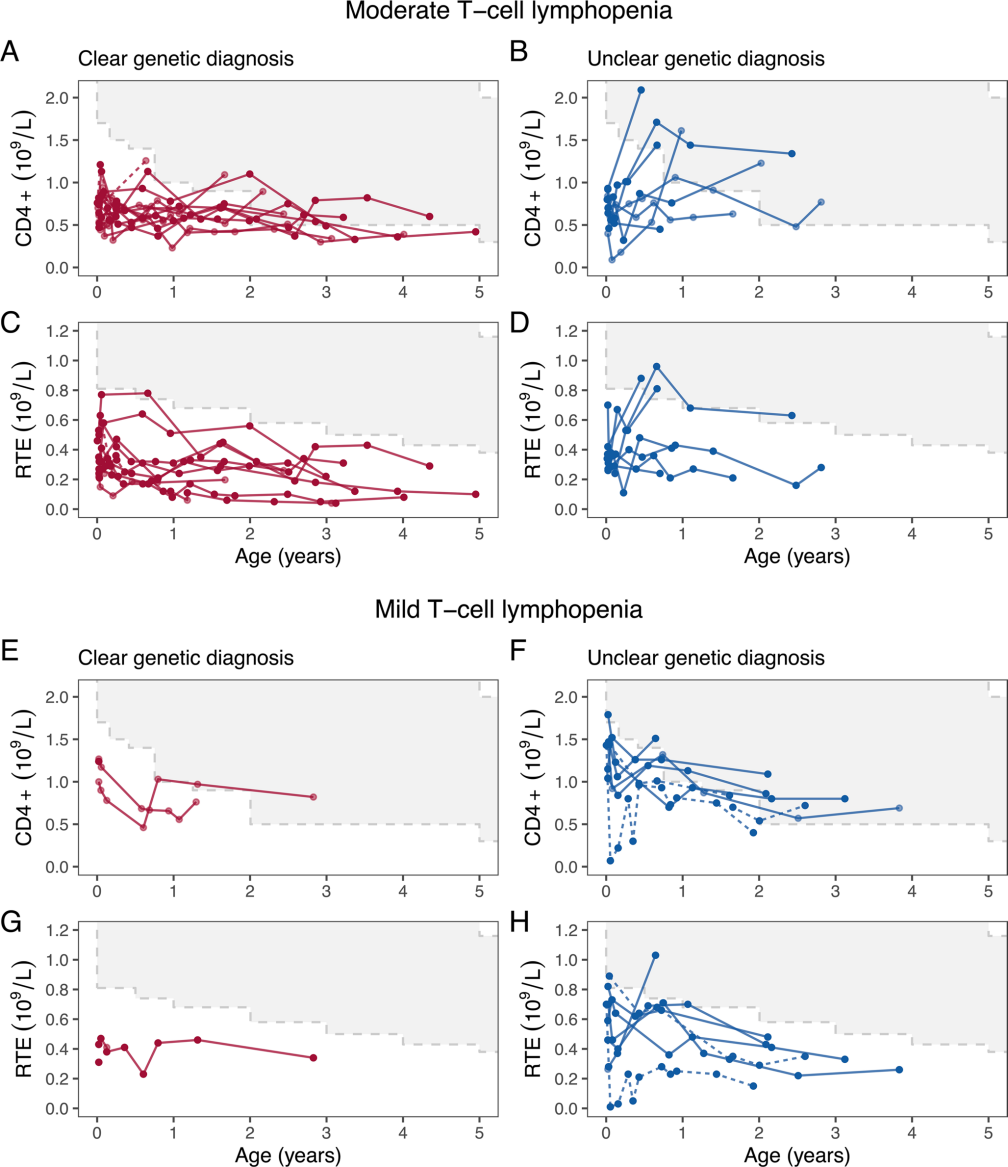
Course of CD4 + and recent thymic emigrant (RTE) counts in patients with moderate and mild T-cell lymphopenia (TCL) in their initial investigations. Patients with a moderate TCL and a clear genetic diagnosis showed relatively stable CD4 + counts, whereas those with an unclear genetic diagnosis often exhibited an increase. Dashed lines represent thoracotomized patients. Grey polygons represent the respective reference ranges

Among the 27 patients with moderate TCL, 14 had a clear genetic diagnosis, 9 had an unclear genetic diagnosis, and 4 had no genetic testing. Patients with a clear genetic diagnosis generally had stable CD4 + T-cell counts, mostly remaining between 0.3 and 1.0 × 10^9^/L throughout the follow-up period. However, their RTE counts were more variable, with a tendency to decrease over time. Among patients with an unclear genetic diagnosis, 6 of 8 with longitudinal data demonstrated an increase in CD4 + counts during the first year of life, and four also showed an increase in RTE counts (Fig. 4A-D).

#### Patients with Mild TCL (CD4 + > 1.0 × 10^9^/L)

Of the 12 patients with mild TCL, a clear genetic diagnosis was found in only 3 cases (22q11.2DS in 2 patients and Trisomy 21 in 1), 8 had an unclear genetic diagnosis and 1 had not undergone genetic testing. In both patients with a clear and unclear genetic diagnosis, CD4 + T-cells counts demonstrated a slight decrease over time, but usually normalized with the decrease of the lower limits of the reference ranges with increasing age. Notably, 1 patient who underwent several thoracotomies, exhibited a more pronounced decrease in CD4 + T-cell counts (Fig. 4E-H).

### Prophylactic Measures

Most patients received prophylactic measures to prevent infections, per criteria detailed in the methods section (see Table 3 for distribution and duration).

**Table 3:** Overview of applied prophylactic measures

Measure	*N* started on (%)	Median age at start (days, range)	*N* taken off (%)^#^	Median duration (months, range)^*^	Time from stopping to last follow-up (months, range)
*Severe TCL (initial CD4 + < 0.3 × 10* ^*9*^ */L)*					
PJP prophylaxis	3 (100)	51 (21 to 57)	1 (33)	21	13
Fungal prophylaxis	2 (67)	33 (8 to 57)	1 (50)	45	10
RSV prophylaxis^†^	2 (67)	44 (14 to 74)	2 (100)	15 (3 to 27)	28 (26 to 31)
IgRT	2 (67)	46 (14 to 77)	0 (0)	NA	NA
*Moderate TCL (initial CD4 + 0.3 to 1.0 × 10* ^*9*^ */L)*					
PJP prophylaxis	20 (71)	24 (7 to 116)	17 (85)	11 (5 to 23)	12 (1 to 44)
Fungal prophylaxis	2 (7)	20 (15 to 25)	2 (100)	8 (5 to 12)	18 (15 to 21)
RSV prophylaxis^†^	1 (4)	45	1 (100)	10	14
IgRT	4 (14)	241 (56 to 932)	2 (50)	4 (4 to 4)	18 (15 to 21)
*Mild TCL (initial CD4 + ≥ 1.0 × 10* ^*9*^ */L)*					
PJP prophylaxis	5 (42)	55 (9 to 161)	5 (100)	6 (0.2 to 8)	19 (12 to 37)

^***#***^only those in whom the measure was actively discontinued are included; patients who continued the respective measure until death are excluded; ^*^in patients actively taken off the respective measures; ^†^seasonal; IgRT – immunoglobulin replacement therapy; *N*, number; *NA*, not applicable; *PJP*, Pneumocystis jirovecii pneumonia; *RSV*, respiratory syncytial virus; *TCL*, T-cell lymphopenia

Among thymic defect patients, 13 of 15 patients (87%) received prophylactic medication, mainly PJP prophylaxis, which was discontinued in all after a median of 12 months (range: 1 week to 23 months). Both AT patients received PJP prophylaxis and IgRT, both continued at last follow-up. Of those with unclear genetic diagnoses, 9 (52%) received prophylaxis (primarily PJP) and discontinued it at a median age of 6 months (range: 5 to 12 months); all but one were diagnosed with iTCL. All 23 patients who stopped PJP prophylaxis had CD4 + counts > 0.5 G/L. In 19 of them (83%), vaccine responses were adequate; for the remaining 4 (17%) data were unavailable or partially insufficient.

In 16 infants (37%), vaccine schedules were adjusted to include a 3rd dose of hexavalent and pneumococcal vaccines at 6 months, in addition to the doses administered at 2 and 4 months, without prior antibody level measurement. Ten patients (23%) showed inadequate specific antibody levels against at least one of three tested vaccines (tetanus, pneumococcal, or *Haemophilus influenzae* type b) after 2 doses and received additional 3rd doses.

Data on live vaccines [MMR(V): measles, mumps, rubella, (varicella)] were available for 37 patients. Four patients did not survive until age 9 months, the recommended MMR(V) dose age per the national schedule. MMR(V) was administered on schedule for 24 patients (65%) and delayed for 7 patients (14%) due to ongoing IgRT in 2, markedly low T-cell counts in 3, hypogammaglobulinemia in 1, or low responses to inactivated vaccines in 1. No adverse effects were reported. Two patients had not yet received live vaccines as they continued regular IgRT on last follow-up.

### Infections

Among patients with severe TCL, the patient with 22q11.2DS experienced frequent viral infections with some leading to exacerbations of her chronic heart failure. She also developed a mediastinitis following multiple thoracotomies. The patient with SGPL1-deficiency required multiple hospitalizations due to infections, including suspected cytomegalovirus colitis, RSV bronchiolitis, and episodes of central line-associated coagulase-negative staphylococcal sepsis. Staphylococcal bacteriemia, possibly related to infected thrombi, may have contributed to her death. In contrast, the patient with 22q11.2 microduplication syndrome had no severe or recurrent infections. Details on infections in the moderate and mild TCL groups are provided in Table E5.

### Hematological Features

Six patients (14%) experienced neutropenia. In 1 patient with AT and 1 with 22q11.2DS, the neutropenia was autoimmune, confirmed by positive antigranulocyte antibodies. The patient with 22q11.2DS also developed thrombocytopenia. The remaining 4 patients (2 with 22q11.2DS, 1 iTCL and 1 without genetic testing) had mild, transient neutropenia that appeared between 2 and 5 weeks of age and lasted for a median of 11 months (range: 8 to 40 months), during which no further diagnostics were pursued. Additionally, there was one case of transient myeloproliferative syndrome immediately after birth in the patient with trisomy 21, but this occurred without a lymphopenia, and TCL in this patient was not considered secondary.

### Factors Associated with Persistent Findings

**Fig. 5:**
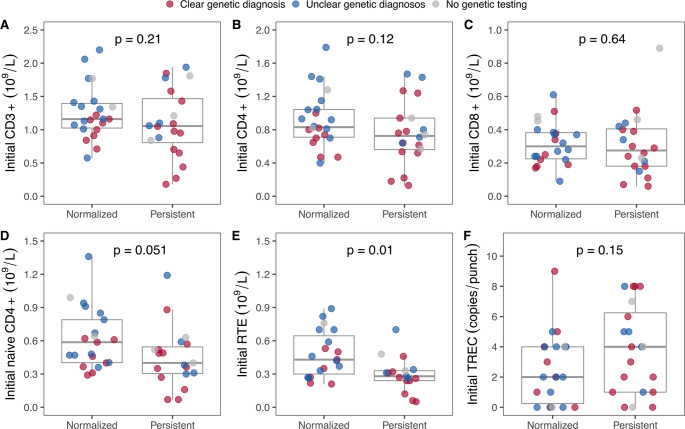
Comparison of initial immunophenotyping and T-cell receptor excision circle (TREC) results in patients with normalization of total T-cells versus those with persistent T-cell lymphopenia. Patients with persistent low T-cells at the last follow-up had lower initial counts of naïve CD4 + T-cells (**D**) and recent thymic emigrants (RTE) (**E**). There was also some tendency for these patients to have lower initial CD4 + T-cell counts (**B**). However, the persistence of low T-cells was not associated with lower initial TREC levels (**F**)

Patients with persistently low T-cell counts at the last follow-up tended to have lower naïve CD4 + T-cell, and RTE counts, and they also tended to have lower CD4 + counts during their initial immunological evaluation. Specifically, no patient with an initial CD4 + count < 0.4 × 10^9^/L, naïve CD4 + count < 0.3 × 10^9^/L or RTE count below 0.2 × 10^9^/L achieved normalization of their total CD3 + T-cells during the follow-up period. There was no association between initial TREC levels and the persistence of TCL or the likelihood of having a clear genetic diagnosis (Fig. 5).

### Non-Immunological Aspects

One of the patients with 22q11.2DS was diagnosed prenatally, while 2 others had been diagnosed with CHD prior to receiving their NBS results, and genetic investigations had already been planned for 1 of them. In the remaining 11 patients, TCL detected by NBS prompted further investigations, leading to the diagnosis of CHD in 4 and hypoparathyroidism in 2. Both patients with AT had no other symptoms at the time of genetic diagnosis; they received neurological evaluations and supportive physiotherapy prompted by NBS.

## Discussion

This study presents a comprehensive retrospective analysis of a well-monitored nationwide cohort of children with non-SCID, non-congenital athymia TCL identified through NBS. A clear genetic diagnosis of an IEI was established in approximately half of the cohort, with thymic defects being the most common [13, 14], followed by AT. A trend was observed in which patients with more severe TCL at initial evaluation were more likely to receive a clear genetic diagnosis. Patients with a clear genetic diagnosis consistently had lower total T-cell and CD4 + counts as well as reduced thymic output throughout their first year of life. They were also less likely to achieve normalization of total T-cell counts compared to those with an unclear genetic diagnosis. Patients with persistently low T-cells on last follow-up typically had lower initial CD4 + counts and thymic output. This suggests that patients with lower initial CD4 + counts, reduced thymic output, and a clear genetic diagnosis are more likely to have persistent TCL. Conversely, patients with milder initial TCL and an unclear genetic diagnosis were more likely to show T-cell normalization over time. These findings provide valuable prognostic insights, which are particularly important for counseling parents and caregivers. While NBS programs offer significant benefits, they can also cause considerable stress due to uncertainty and limited information [20, 21]. Although these findings may not apply to all patients, they offer critical guidance for predicting outcomes and counseling families.

In addition to differences in T-cell trajectories, variations in prophylactic measures and infection rates were also observed. Patients with severe TCL showed limited T-cell improvement and seldom discontinued prophylactic measures. This group also experienced the only opportunistic infection, specifically cytomegalovirus colitis. Patients with moderate TCL and a known thymic defect generally maintained stable T-cell counts, allowing for discontinuation of prophylactic measures by a median age of 12 months, and at the latest by 2 years. Those with moderate TCL and an unclear genetic diagnosis often experienced T-cell recovery, enabling earlier cessation of prophylactic measures, a pattern similarly observed in patients with mild TCL. However, patients with moderate TCL and AT required long-term prophylactic measures. Beyond the single case of cytomegalovirus infection, no other opportunistic infections occurred, and no unusual infections were reported in patients without indwelling catheters or intensive care hospitalizations due to comorbidities. Although the observational nature of this study limits our ability to quantify the impact of prophylactic interventions, our data suggest that infection prevention with limited interventions – such as PJP prophylaxis, tailored administration of live vaccines, fungal prophylaxis, seasonal RSV vaccination, and IgRT in selected cases – is both feasible and safe for this population. However, further studies comparing criteria for initiation and discontinuation of prophylaxis are needed to develop evidence based guidelines and optimize care of patients with non-SCID, non-congenital athymia TCL.

Our findings offer valuable insights for managing and counseling similar patients identified through NBS. Early and comprehensive genetic evaluation provides prognostic information, even in cases where no clear genetic diagnosis is reached. Furthermore, many patients with a clear genetic diagnosis also have non-immunological actionable findings, such as CHD or hypoparathyroidism in some patients with 22q11.2DS, or undiagnosed neurological disease in AT. A multidisciplinary approach by an experienced team ensures not only infection prevention but also maximizes the benefits of early diagnosis of these conditions.

This study is the first to investigate the non-SCID TCL population in such detail, with longitudinal data starting from birth. While previous studies have reported TCL resolution in a subset of NBS patients [13, 14], our findings offer a more nuanced understanding of prognostic outcomes based on initial immunological findings and the presence or absence of a genetic diagnosis. We also provide detailed long-term follow-up data, including immunophenotyping beyond the first year of life. However, there are limitations to our work: the cohort represents only a subset of patients with rare conditions potentially identifiable via TREC screening [5], limiting our ability to generalize for all TCL-related conditions and the observational design of the study restricts our capacity to quantify the benefits of NBS and prophylactic interventions. As our understanding of genetic diseases and immunological phenotypes evolves, some cases currently categorized as “unclear genetic diagnosis” may ultimately be reclassified. However, patients generally had the mild TCL a high likelihood of recovery, so we do not believe this introduces significant bias.

In summary, this study provides extensive follow-up data on a cohort of patients with non-SCID, non-congenital athymia TCL. Even with limited prophylactic measures, overall outcomes were favorable, with no clearly TCL-associated lethal infections, very few opportunistic infections, and rare severe viral infections. These findings will inform the management of similar patients and provide valuable support for counselling their families.

## Supplementary Information

Below is the link to the electronic supplementary material.

Supplementary Material 1

## Data Availability

The datasets generated and analyzed during the current study are available from the corresponding author on reasonable request.

## Abbreviations

22q11.2DS: 22q11.2 deletion syndrom
AT: Ataxia–telangiectasia
CD: cluster of differentiation
CHD: congenital heart disease
DBS: dried blood spots
HSCT: hematopoietic stem cell transplantation
IEI: inborn errors of immunity
IgRT: immunoglobulin replacement therapy
iTCL: idiopathic T–cell lymphopenia
KREC: kappa–deleting recombination excision circles
NBS: newborn screening
MMR(V): measles, mumps, rubella, (varicella)
PJP: Pneumocystis jiroveci pneumonia
RSV: respiratory syncytial virus
RTE: recent thymic emigrant
SCID: severe combined immunodeficiency
TCL: T–cell lymphopenia
TREC: T–cell receptor excision circle
TT: thymus transplant
VUS: variants of unknown significance
WES: whole–exome sequencing
