# Supplementary material for: Natural History of Swiss Infants with Non-SCID T-cell Lymphopenia Detected by Newborn Screening: A Cohort Study
Source: J Clin Immunol. 2025 Nov 25;45(1):166. doi: 10.1007/s10875-025-01945-4 (PMC12647285; doi:10.1007/s10875-025-01945-4)
Supplement: Supplementary file 1 — Supplementary Material 1 [file 10875_2025_1945_MOESM1_ESM.pdf]

Natural history of Swiss infants with non-SCID T-cell lymphopenia detected by newborn screening: a cohort study

Soomann *et al*

Supplementary material

Tables

Table S1. Packages used for data analysis in R version 4.2.2

Type	Packages
Base packages	grid, stats, graphics, grDevices, utils, datasets, methods, base
Additional packages	reshape2 1.4.4, ggbeeswarm 0.7.1, ggbreak 0.1.2, deeptime 1.0.1, ggpubr 0.6.0, ggthemes 4.2.4, readxl 1.4.2, lubridate 1.9.2, forcats 1.0.0, stringr 1.5.1, dplyr 1.1.0, purrr 1.0.1, readr 2.1.4, tidyr 1.3.0, tibble 3.1.8, ggplot2 3.4.1, tidyverse 2.0.0

Table S2. Statistical methods

Category	Variables	Used methods
Descriptive	continuous	median, minimum, maximum
	categorical	counts, percentages
Interferential	categorical	Fisher's exact test, χ^2 test
	categorical and continuous	Mann-Whitney U-test
	categorical and time-to-event data	Kaplan-Meier estimator for overall survival probabilities*<br>Log-rank test<br>Cox-Regression<br>Hazard ratios

** Throughout the time-to-event analysis normalization of cell counts was treated as the event of interest, with those not reaching values within the local reference ranges, deceased, or lost to follow-up being right-censored on the day of their last documented lymphocyte subset analysis.*

Table S3. Overview of patients with secondary T-cell lymphopenia

N	Gestational age at birth (weeks)	Likely cause of secondary TCL	Final findings	Age at normalization (days)	Outcome	Age at death (days)	Cause of death
1	25 0/7	severe prematurity	TREC normalized	83	alive	-	-
2	26 0/7	severe prematurity	TREC normalized	15	alive	-	-
3	38 0/7	critical illness with pancytopenia of unknown cause	full blood count normalized	109	alive	-	-
4	39 1/7	critical illness due to a congenital heart disease	lymphocyte subsets normalized	7	alive	-	-
5	40 5/7	critical illness with intestinal perforation and pancytopenia	lymphocyte subsets normalized	28	alive	-	-
6	40 0/7	in utero azathioprine exposure and reduced maternal TPMT activity	lymphocyte subsets normalized	75	alive	-	-
7	35 2/7	Langerhans cell histiocytosis*	persistent low T-cells and low thymic output	-	deceased	19	septic shock in neutropenia
8	38 1/7	critical illness due to a congenital heart disease*	persistent low T-cells and low thymic output	-	deceased	49	multiorgan failure

** – no abnormalities in known inborn errors of immunity genes in trio-exome sequencing; N – number; TCL – T-cell lymphopenia; TPMT – thiopurine methyltransferase; TREC – T-cell receptor excision circles*

Table S4. Diagnoses, course of immunological findings and details on death in the deceased

Nr	Final diagnosis	TCL course	Age at death	Cause of death
1	22q11.2 deletion syndrome	persistent	5 months	chronic heart failure
2	22q11.2 deletion syndrome	persistent	3 years	chronic heart failure
3	SGPL1-deficiency	persistent	9 months	multifactorial schock, pulmonary embolism, bacteriemia with coagulase negative Staphylocci
4	Trisomy 21	persistent	1.5 months	pulmonary hypoplasia
5	Unknown syndromal disease with TCL	persistent	5 days	redirection of care

SGPL1– Sphingosine-1-Phosphate Lyase 1; TCL – T-cell lymphopenia

Table S5. Infection experienced by patients with moderate and mild T-cell lymphopenia

**Patient group**	**Final diagnosis**	**Infections**	**Organism**
Moderate T-cell lymphopenia			
	Ataxia-telangiectasia	frequent viral respiratory infections	
	22q11.2 deletion syndrome	sepsis acquired while on intensive care due to cardiac disease	*Serratia marcescens*
	22q11.2 deletion syndrome	severe bronchiolitis	respiratory syncytial virus
	22q11.2 deletion syndrome	pyelonephritis	*Escherichia coli*
	22q11.2 deletion syndrome	otitis media	
	22q11.2 deletion syndrome	pneumonia	
	Idiopathic T cell lymphopenia	pyelonephritis	*Escherichia coli*
	Idiopathic T cell lymphopenia	frequent viral respiratory infections	
	Unclear syndromal disease with T cell lymphopenia	ischemic colitis	
	Unclear syndromal disease with T cell lymphopenia	suspected early-onset neonatal sepsis	
	No genetic testing	suspected early-onset neonatal sepsis	
	No genetic testing	severe bronchiolitis<br>otitis media	respiratory syncytial virus
Mild T-cell lymphopenia			
	KBG syndrome	gastroenteritis requiring hospitalization<br>recurrent otitis media	rotavirus

Figures

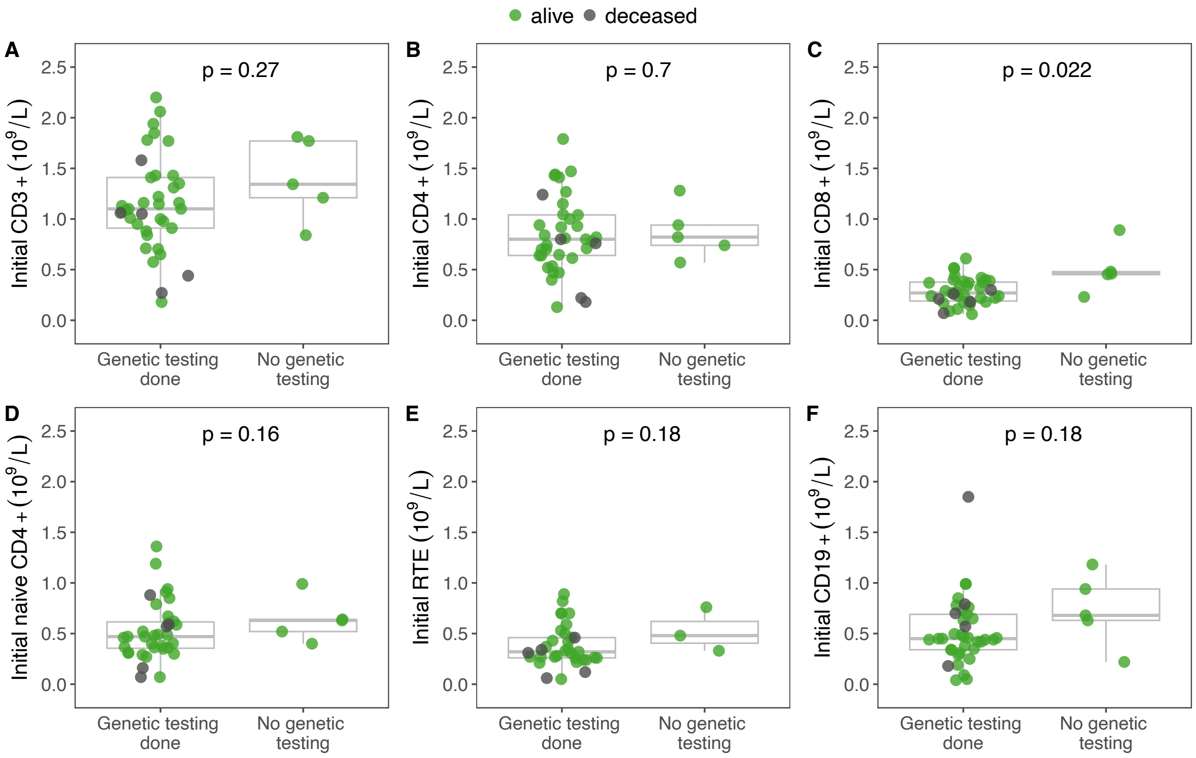

Figure S1. Comparison of lymphocyte subset results in those who underwent genetic testing und those who did not. Except for CD8+ T-cells, there were no statistically significant differences in the total number of CD3+ T-cells or any of the T-cell subsets. *RTE – recent thymic emigrant; TREC – T-cell receptor excision circle*

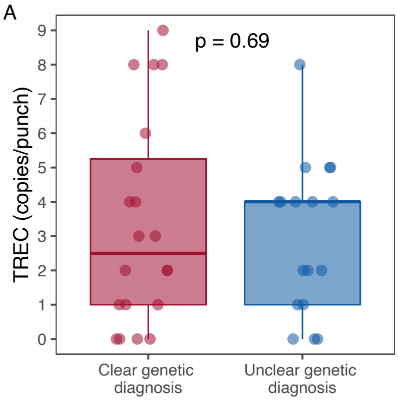

Figure S2. Initial TREC levels and establishing a genetic diagnosis. The distribution of TREC values was similar in those with a clear genetic diagnosis and those without. *TREC – T-cell receptor excision circle.*

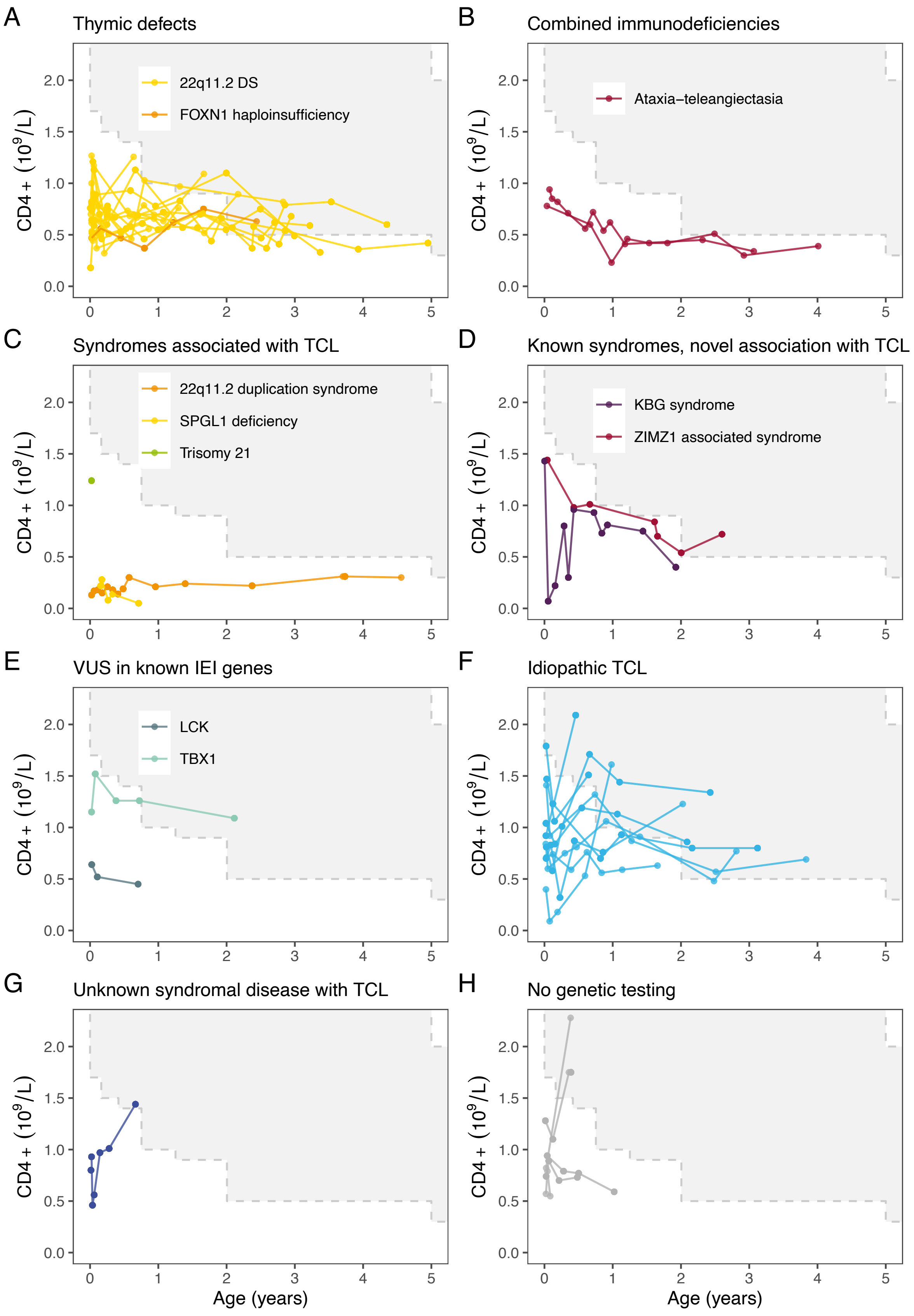

Figure S3. Course of CD4+ counts in individual patients grouped by diagnostic subcategory. Grey polygons represent the reference range. Whereas patients with thymic defects showed relatively stable CD4+ counts, patients with AT showed a continuous decline. CD4+ counts often ameliorated in time in patients with idiopathic TCL. *IEI – inborn errors of immunity; TCL – T-cell lymphopenia; VUS – variant of unknown significance*

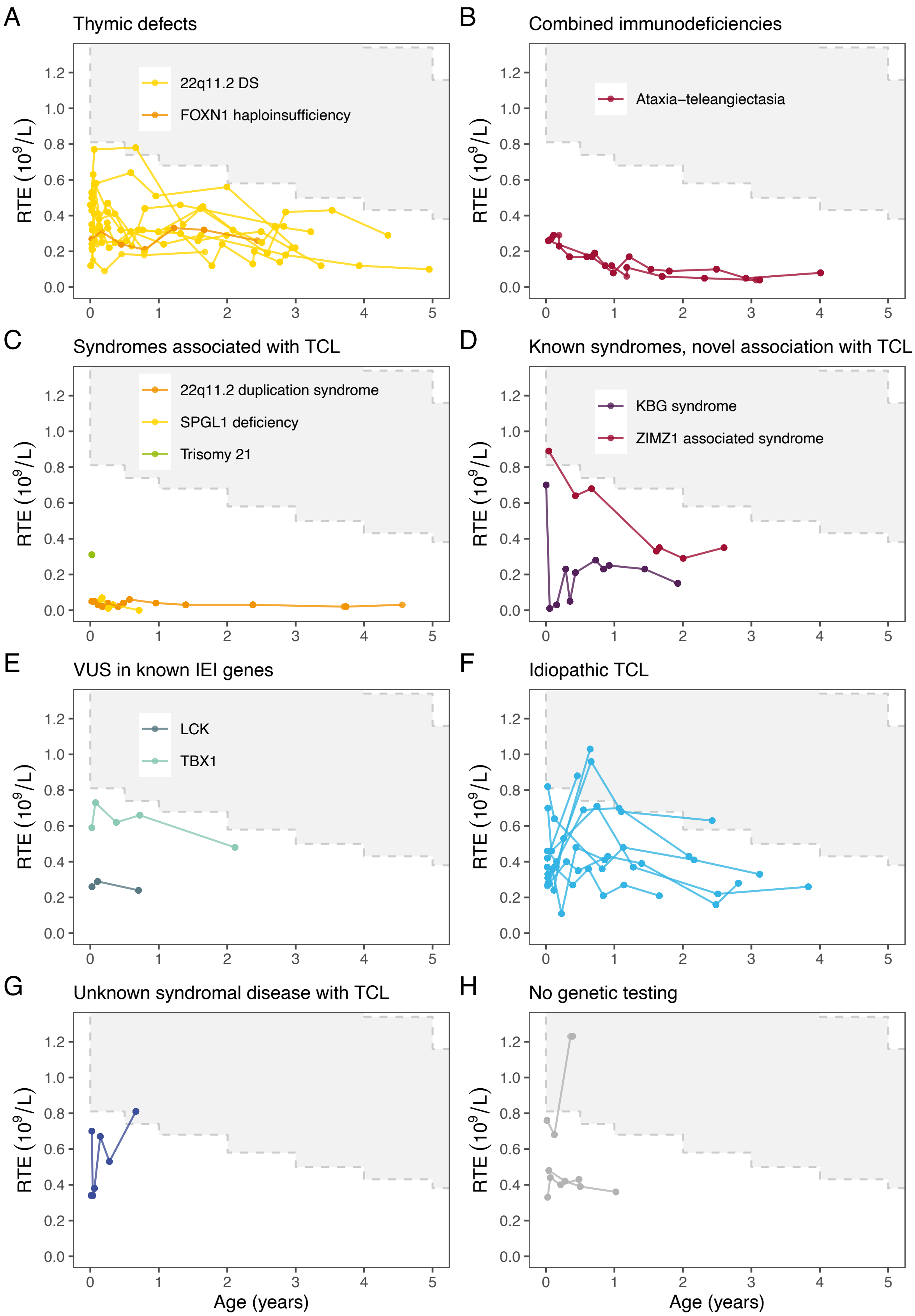

Figure S4. Course of recent thymic emigrant (RTE) counts in individual patients grouped by diagnostic subcategories. Grey polygons represent the reference range. *IEI – inborn errors of immunity; RTE – recent thymic emigrant; TCL – T-cell lymphopenia; VUS – variant of unknown significance*
